# Insertions and deletions in the RNA sequence–structure map

**DOI:** 10.1098/rsif.2021.0380

**Published:** 2021-10-06

**Authors:** Nora S. Martin, Sebastian E. Ahnert

**Affiliations:** ^1^ Theory of Condensed Matter Group, Cavendish Laboratory, University of Cambridge, JJ Thomson Avenue, Cambridge CB3 0HE, UK; ^2^ Sainsbury Laboratory, University of Cambridge, Bateman Street, Cambridge CB2 1LR, UK; ^3^ Department of Chemical Engineering and Biotechnology, University of Cambridge, Philippa Fawcett Drive, Cambridge CB3 0AS, UK; ^4^ The Alan Turing Institute, British Library, Euston Road, London NW1 2DB, UK

**Keywords:** genotype–phenotype map, sequence–structure map, RNA secondary structure, robustness

## Abstract

Genotype–phenotype maps link genetic changes to their fitness effect and are thus an essential component of evolutionary models. The map between RNA sequences and their secondary structures is a key example and has applications in functional RNA evolution. For this map, the structural effect of substitutions is well understood, but models usually assume a constant sequence length and do not consider insertions or deletions. Here, we expand the sequence–structure map to include single nucleotide insertions and deletions by using the RNAshapes concept. To quantify the structural effect of insertions and deletions, we generalize existing definitions for robustness and non-neutral mutation probabilities. We find striking similarities between substitutions, deletions and insertions: robustness to substitutions is correlated with robustness to insertions and, for most structures, to deletions. In addition, frequent structural changes after substitutions also tend to be common for insertions and deletions. This is consistent with the connection between energetically suboptimal folds and possible structural transitions. The similarities observed hold both for genotypic and phenotypic robustness and mutation probabilities, i.e. for individual sequences and for averages over sequences with the same structure. Our results could have implications for the rate of neutral and non-neutral evolution.

## 1. Introduction

The genotype–phenotype relationship is a ‘cornerstone’ [1] of molecular evolution because it captures the structural and functional consequences of mutations. These mutations can include substitutions as well as insertions and deletions and are the source of variation. Therefore, genotype–phenotype maps are needed in models for the emergence of new structures and functions [2,3] as well as in models of neutral evolution, when the sequence accumulates mutations without any structural and functional changes [4,5]. Sequence–structure maps are a specific case within the more general framework of genotype–phenotype maps: here sequences are treated as genotypes and structures as phenotypes.

In practice, a sequence–structure map is interesting if the molecular structure is functionally important and its large-scale analysis is feasible if a fast computational prediction method exists. The secondary structure of RNA sequences fulfils these two criteria [6] and has therefore become one of the best-studied sequence–structure maps (recent examples are [7–12]). The secondary structure is the pattern of base pairs between nucleotides, usually not including pseudoknots. This base pairing pattern can be described on different levels of detail [13–15]. In sequence–structure map research, the ViennaRNA package [16] is most commonly used [7,17,18]. This program returns structures at the most detailed level, where the predicted structure has a resolution of individual base pairs and is based on a minimum-free-energy (*mfe*) criterion.

**Figure 1.**
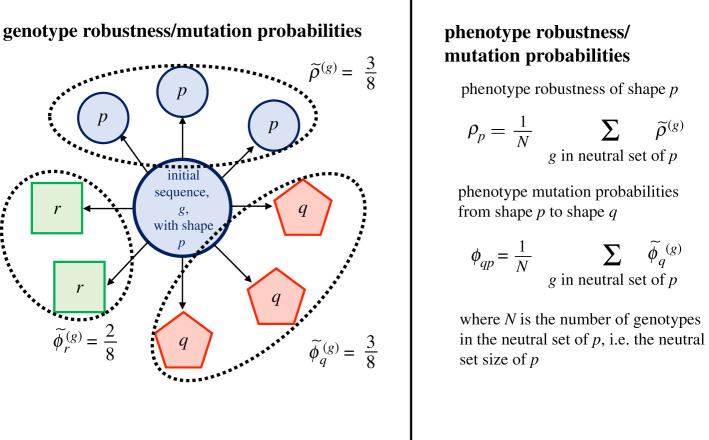
Schematic of robustness and mutation probabilities. Genotypic quantities (left): for a given sequence *g* (central circle) folding into shape *p*, this figure shows the entire mutational neighbourhood, i.e. the outcomes of all possible substitutions (arrows). The genotype robustness is 3/8 since three out of eight substitution are neutral (i.e. also fold into *p*). For non-neutral mutations, the genotype mutation probabilities ${\tilde{\phi }}_{q}^{\left(g\right)}$ are calculated from the number of times each non-neutral shape *q* is found in the mutational neighbourhood. *q* can be any structure except the unfolded state. If a shape *t* does not exist in the mutational neighbourhood, ${\tilde{\phi }}_{t}^{\left(g\right)}=0$. Throughout the text, we use a tilde (∼) for these genotypic quantities. Phenotypic quantities (right): phenotype robustness and mutation probabilities are calculated by taking the average of the corresponding genotypic quantities over all sequences in the neutral set of a given shape. These definitions follow [3,6,18], but with a different notation.

Most studies of the RNA sequence–structure map have focused on sets of sequences of fixed length [7,15,17,18]. For these, results exist on both the global statistics of sequences and structures [17,19,20] and the structural effects of mutations on a local level [6,18,21], i.e. the mutational neighbourhood (illustrated in figure 1). On a global level, it was found that there is usually a number of sequences folding into the same structure [17,19] and these sets are referred to as *neutral sets* [22]. On the local level, previous research has focused on single-nucleotide substitutions [6,7,18,21]. Their structural effect can either be *neutral* if there is no structural change or *non-neutral* otherwise. Neutral mutations are quantified by robustness: *genotype robustness*, ${\tilde{\rho }}^{\left(g\right)}$, measures what fraction of substitutions are neutral for a given sequence *g* [6] (figure 1). Non-neutral mutations are summarized by genotype mutation probabilities, here referred to as ${\tilde{\phi }}_{q}^{\left(g\right)}$, which describe how frequently an alternative structure *q* appears after substitutions on sequence *g*, as illustrated in figure 1 (defined as $\phi_{qg}^{\left(\mathrm{local}\right)}$ [18]). In addition to these sequence-specific definitions, which we denote by a tilde (∼) in this paper, the quantities are often averaged over all sequences in a neutral set, i.e. over sequences with the same structure. These structure-specific quantities are referred to as *phenotype robustness* [6], *ρ*_*p*_, and *phenotype mutation probability* *ϕ*_*qp*_ [3,18] (both defined in figure 1). These phenotypic quantities describe the mean effect of mutations for a population going through a period of neutral evolution [3].

With these concepts, the properties of mutational neighbourhoods have been quantified in detail [6,18]: for example, Greenbury *et al.* [18] found that similar sequences are more likely to have the same structure than random sequences, described how this decreases the structural diversity in mutational neighbourhoods and coined the term *genetic correlations*. A deeper understanding of mutational neighbourhoods has been derived from thermodynamic considerations. For non-neutral mutations, Ancel & Fontana [23] found that it is common for structures that emerge after a non-neutral mutation to have existed as an energetically suboptimal structure with high Boltzmann frequency before the mutation. They therefore conclude that the Boltzmann ensemble is linked to the effect of substitutions and called this principle *plastogenetic congruence* [23].

Thus substitutions have been well studied, but so far insertions or deletions (*indels*) are missing from this picture even though they occur frequently in biological databases [24]. Indels are important because neither neutral nor non-neutral evolution takes place at fixed sequence length: different protein folds or functional RNAs can have different sequence lengths. Thus, the non-neutral evolution of new structures must include sequence length variation. Furthermore sequence families, for example in Pfam [25] and Rfam [26], often contain insertions, deletions and sequence length changes and so sequence length changes occur in structurally nearly-neutral evolution as well. For proteins, short indels have been analysed in some detail due to their frequent occurrence [27,28], even though the effect can be more complex in proteins due to potential reading frame shifts [28]. Among reading-frame-preserving indels, neutral mutations are preferentially found in specific structural contexts [27–29]. Together with additional information about the proteins, this can be used to predict if a short indel is likely to be neutral [28,30]. For RNA, the few studies that have included indels either measure robustness only for specific example structures [31], focus on insertion–deletion pairs which do not change the sequence length [32], or do not quantify the sequence–structure map properties, but instead model specific evolutionary processes [33]. The reason for this gap is a fundamental restriction in the commonly used mfe structure representation: if structures are defined on a single-base-pair resolution, then structures of different lengths cannot be treated in a single framework.

**Figure 2.**
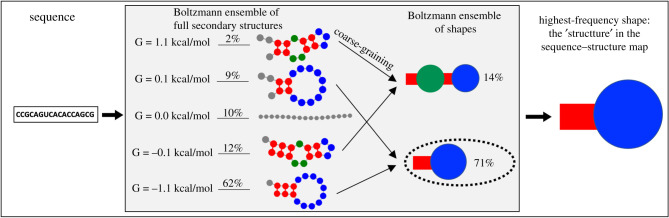
Schematic of the shapes sequence–structure map: for the example input sequence CCGCAGUCACACCAGCG, the shapes framework [38] represents the Boltzmann distribution of different folding possibilities in terms of coarse-grained shapes instead of full secondary structures, as illustrated in the central box. Each shape represents one or more full secondary structures. Our sequence–structure map is defined from this framework as follows: we start with a sequence as an input. Then we calculate the Boltzmann ensemble of shapes as described in §2.5 and illustrated in the central box. Finally, a single shape, the shape with the highest Boltzmann frequency, is chosen as the output structure in the sequence–structure map. In this example, this is the stem-loop shape ‘[]’ with a Boltzmann frequency of 71% (the shape notation uses square brackets, as described in [38]). Shapes other than the most frequent shape in the Boltzmann ensemble are only considered in our analysis of plastogenetic congruence. Throughout the paper, we will refer to the abstract, coarse-grained structures defined by RNAshapes as shapes or structures, and the detailed structures from minimum-free-energy (*mfe*) predictions as *full secondary structures*. Structures are drawn with forna [39], the shape illustrations are inspired by Meyers *et al*. [40] and all structures with Boltzmann frequencies greater than 1% are included in the schematic.

To address this issue and to include indels in the established framework, we use the RNAshapes concept [34,35], which captures RNA secondary structure in a more abstract and coarse-grained way than the commonly used detailed full secondary structure descriptions. In addition, the concept has further advantages: Dingle and co-workers [36] argue that minor structural differences should not be included in the model and are thus the first to use RNAshapes in a sequence–structure map context. An additional advantage of the coarse-grained structures, or *shapes*, is that they better capture the Boltzmann ensemble of suboptimal structures [35]: secondary structures are reported in less detail when the Boltzmann states are abstract shapes rather than full secondary structures, and so individual Boltzmann frequencies are higher [37] (schematic in figure 2). This is important because in a full secondary structure description, the Boltzmann frequency of even the most frequent structure is often low [41]. In our analysis, we will refer to the abstract, coarse-grained structures defined by RNAshapes as shapes or structures, and the detailed structures from minimum-free-energy (*mfe*) predictions as full secondary structures.

In this paper, we use the shapes framework to analyse mutational neighbourhoods in which sequence lengths change by single-nucleotide insertions and deletions. This means we will study three mutational neighbourhoods per sequence, one by insertion, one by deletion and one by substitution and thus compare the structural effect of three types of mutations. For this comparison, we extend the existing definitions for genotype and phenotype robustness, and genotype and phenotype mutation probability to insertions and deletions. The first part of the paper focuses on neutral mutations: we start with the sequence-dependence of genotype robustness within neutral sets. Next, we focus on differences in phenotype robustness between neutral sets. In the second part of the paper, we consider non-neutral mutations: we analyse the sequence-dependence of genotype mutation probabilities within neutral sets. Then, we compare phenotype mutation probabilities for different target shapes.

## 2. Methods

### 2.1. Definition of mutational neighbourhoods

Genotype robustness and genotype mutation probabilities for substitutions are based on the definition of a mutational neighbourhood [6,18] (as illustrated in figure 1). Therefore, these quantities can be extended to insertions and deletions if we define the mutational neighbourhood by insertion and deletion for a given sequence.

For substitutions, the mutational neighbourhood is defined as follows: it contains all sequences which can be generated from the start sequence by applying any single-nucleotide substitution [21]. Because each substitution leads to a distinct new sequence, each sequence in the mutational neighbourhood by substitution is unique. For deletions and insertions on the other hand, there are cases where two distinct insertions/deletions effect the same sequence change: for example, for sequence UAAC, deletions at positions 2 or 3 would both result in the sequence UAC. In our study, these two deletions will be considered separate mutational neighbours since they were generated by distinct deletions. Thus UAC would exist twice in the mutational neighbourhood. This definition emulates a uniform insertion or deletion probability.

Since the purpose of this paper is to compare the effect of substitutions, insertions and deletions, we will treat each of these types of mutational neighbourhoods individually and thus compute robustness and mutation probabilities for substitutions, insertions and deletions separately. Substitutions will be considered the baseline for all comparisons since they are already well understood.

### 2.2. Robustness/mutation probability definitions

Once the mutational neighbourhood for each sequence is defined, genotype robustness and mutation probabilities for a specific sequence can be computed as illustrated in figure 1. Phenotype robustness and mutation probabilities follow from these genotypic quantities once we define a neutral set: here, we only study mutations applied to sequences of fixed sequence length *L* = 30. Therefore, our neutral sets are sets of *L* = 30 sequences which share the same structure *p*, i.e. the established definition of neutral sets [1]. Once the neutral set is defined, we can compute phenotype robustness and mutation probabilities as averages over the neutral set (as shown in figure 1). For computational reasons, we approximate the averages over all sequences in the neutral set by averages over 500 sequences in the given neutral set, as detailed in §2.6. The sample size is discussed in the electronic supplementary material.

### 2.3. Parameters

The shape abstraction level [35] was chosen based on thermodynamic considerations: since single base pairs at the end of stacks only contribute a single stacking term, a desirable property of the shape abstraction would be that breaking base pairs at the end of long stacks is considered a minor change and not a change of coarse-grained structure. This requirement is not satisfied by level-1 abstractions, as defined in [38], because for example breaking the outermost base pair in (((…))) would lead to a change of coarse-grained structure. Therefore, we use level 2. In the level-2 shape abstraction, all paired and unpaired segments of the structure are recorded except the unpaired segments in multiloops, exterior loops and hairpin loops [38].

We start with sequences of a single fixed sequence length in this analysis and observe the effect of single-nucleotide substitutions, deletions and insertions. This length was set to *L* = 30 nucleotides to strike a balance between structural complexity and computational feasibility, similar to [6].

### 2.4. Sequence–structure map definition

A sequence–structure map translates each sequence to a single structure. The shape framework also takes a sequence as an input, but as an output it returns a list of shapes and their Boltzmann frequencies [35]. Therefore, we need to define how these computed Boltzmann ensembles are used for the sequence–structure map. Here we choose the shape with the highest Boltzmann frequency. This definition mirrors the more commonly used [3,4,6–8,15,18–21,42–44] mfe sequence–structure map: the mfe structure is always the most frequent structure in the Boltzmann ensemble when the Boltzmann distribution is expressed in terms of full secondary structures instead of shapes.

One additional constraint was used to ensure that the highest shape frequency is not a close tie between two shapes: sequences for which the Boltzmann frequency of the most frequent shape is not more than 1.1 × the Boltzmann frequency of the second-most-frequent shape are treated as non-folding. In addition, all sequences for which the most frequent shape is the unfolded structure with no base pairs are treated as non-folding.

### 2.5. Shapes computation

Instead of using the existing RNAshapes [35] program, we use ViennaRNA [16,45–47] and compute shape probabilities by adding up Boltzmann probabilities for the suboptimal structures within a free energy range of *G*_mfe_ ≤ *G* < *G*_mfe_ + 15*k*_*B*_*T*, where *G*_mfe_ is the free energy of the mfe structure. Thus, full secondary structures are included in the calculation if they have a Boltzmann frequency of more than ≈3 × 10^−7^ times the Boltzmann frequency of the mfe structure: exp (− (*G*_mfe_ + 15*k*_*B*_*T*)/*k*_*B*_*T*) ≈ 3 × 10^−7^ × exp (− (*G*_mfe_)/*k*_*B*_*T*). The high free energy range of 15*k*_*B*_*T* was chosen for accuracy, but the exact value is arbitrary. Our calculation uses the same principles as figure 2, but includes full secondary structures up to much lower frequencies.

Calculating shape frequencies in ViennaRNA is not as accurate as the existing RNAshapes [35] program, but it is faster for sequences of length *L* = 30 and the differences in computed shape probabilities are small (data in electronic supplementary material).

Defaults were used for all parameters with one exception: lonely base pairs were not permitted, following the convention in the RNAshapes framework [35].

### 2.6. Sequence sample

For a full systematic analysis, the two genotypic quantities—genotype robustness and genotype mutation probabilities—could be computed for every possible sequence *g*. However, there are 4^30^ ≈ 10^18^ sequences of length *L* = 30 and so this would be infeasible. Variations in genotypic quantities will be partly due to specific sequence-dependent effects and partly due to more general shape-dependent ones. We will focus on the first aspect and study variations within neutral sets, i.e. for a single shape at a time, by computing each genotypic quantity for 500 sequences per neutral set and then making comparisons within each neutral set.

The phenotypic quantities are defined as average values over neutral sets. However, individual neutral sets can contain large numbers of sequences and therefore an exact calculation of the average value is infeasible. Thus, we estimate the phenotypic quantities based on the same fixed sample of 500 sequences per neutral set (the effect of the sample size and sampling method are discussed in the electronic supplementary material).

For these calculations, we need a list of shapes and a sequence sample of 500 sequences for the neutral set of each shape. These data were generated using the following methods:

1. **Listing neutral sets:** we listed all full secondary structures for sequence length *L* = 30 and computed their shapes. This gives a full list of possible shapes.
2. **Initial sequences for each shape:** to generate 10 start sequences per shape for the site-scanning method [49], we used ViennaRNA’s inverse folding on randomly selected full secondary structures belonging to the specified shape. This is repeated up to 5000 times until a valid start sequence is found. Only shapes for which ten start sequences are identified using this method are included in the further analysis.
3. **Full sequence sample for each shape:** starting from each initial sequence, a larger sequence sample of size 50 was found using the site-scanning method [49] for a walk length of 50 × *L*. The site-scanning method uses a specific random walk to generate a diverse sequence sample [49]. We made two modifications compared to the original method: first, we used shapes instead of mfe structures. Second, in order to sample from neutral sets and not only from connected neutral components, we included base pair swaps in addition to substitutions: if a site was paired with another site in the lowest-energy full secondary structure per shape, base pair swaps were used instead of simple substitutions. Base pair swaps will remove one key reason for the fragmentation of neutral sets [8].

With these methods, we obtain a sample of 227 shapes and 500 sequences from the neutral set of each of these shapes. In the electronic supplementary material, we perform several tests to check if these 500 sequences per neutral set are in some way a biased sample from the neutral set and do not find any problems. In addition, we analyse if the sample size influences the results (also in the electronic supplementary material). All results in this paper are based on this sequence sample, except our phenotype frequency estimates.

Phenotype frequencies are estimated following [19]: a random sample of 10^7^ sequences was generated and their shapes computed. The shape frequencies in this sample were used as estimates for their phenotype frequency, i.e. the fraction of sequences folding into the given shape, as defined in [18]. If fewer than 10 instances of the shape are found, the phenotype frequency cannot be estimated reliably and so no estimate was computed. All shapes in the random sequence sample are among the 227 shapes included in this analysis. This confirms that no shape with high phenotype frequency is missed in the construction of our sample.

## 3. Results

### 3.1. Genotype robustness

**Figure 3.**
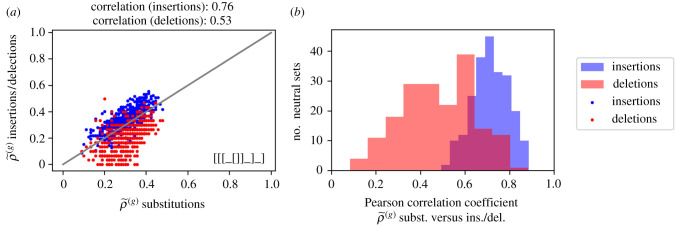
Genotype robustness (${\tilde{\rho }}^{\left(g\right)}$) variation per shape. (*a*) Example for neutral set of shape ‘[[[_[]]_]_]’: for 500 sequences in the neutral set, ${\tilde{\rho }}^{\left(g\right)}$ for insertions (blue)/deletions (red) is plotted against ${\tilde{\rho }}^{\left(g\right)}$ for substitutions. The grey line indicates a one-to-one correspondence. We find that the different types of ${\tilde{\rho }}^{\left(g\right)}$ are correlated, as indicated by the Pearson correlation coefficients above the plot. (*b*) The Pearson correlation coefficient between ${\tilde{\rho }}^{\left(g\right)}$ for substitutions and ${\tilde{\rho }}^{\left(g\right)}$ for insertions/deletions is computed for each neutral set. For each neutral set, the correlation is calculated for 500 sequences from the given neutral set. Cases where ${\tilde{\rho }}^{\left(g\right)}$ shows no variation over the neutral set (for example, zero everywhere) are not included.

The genotype robustness of a sequence is the fraction of mutations which do not lead to a structural change [6]. Differences in robustness will be partly due to specific sequence-dependent effects and partly due to more general shape-dependent ones. We will focus on the first aspect and study genotype robustness within neutral sets, i.e. for a single shape at a time. In figure 3*a*, the genotype robustness to insertions and deletions is plotted against the genotype robustness to substitutions for the 500 sequences we collected from the neutral set of shape ‘[[[_[]]_]_]’. The three types of robustness are correlated.

The analysis was repeated for all neutral sets and the results are summarized as Pearson correlation coefficients in figure 3*b* (full data for further shapes are shown in the electronic supplementary material). These values confirm that the robustness to different mutations is correlated to some extent for most neutral sets. The correlation is higher for substitutions/insertions than for substitutions/deletions. One reason could be the fixed size of mutational neighbourhoods and the resulting discreteness of genotype robustness: there are only *L* deletions for each sequence, compared to 3 × *L* substitutions and 4 × (*L* + 1) insertions. This number is the denominator of the genotype robustness value and therefore robustness to deletions is measured on the most coarse-grained scale. In addition, for 48 neutral sets the robustness to deletions does not vary at all and is zero for all sequences in our sample. It is clear that no correlation can be computed in these neutral sets and these values are not included in the plot. Nevertheless, we can conclude that sequences that are robust to one type of mutation also tend to be robust to other types of mutations.

### 3.2. Phenotype robustness

**Figure 4.**
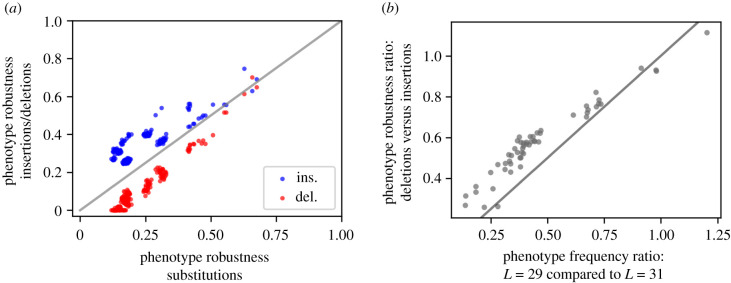
Phenotype robustness (*ρ*_*p*_) variation between shapes. (*a*) The shape-specific phenotype robustness *ρ*_*p*_ for insertions (blue)/deletions (red) is plotted against *ρ*_*p*_ for substitutions for each of the 227 shapes. We find that the different types of *ρ*_*p*_ are correlated. (*b*) The phenotype frequency ratio (*L* = 29 compared to *L* = 31) is compared to the phenotypic robustness ratio (deletions versus insertions). Shapes are only included in this plot if phenotype frequency estimates (for *L* = 29 and *L* = 31) were found using sequence-sampling; see methods §2.6. This is the case for 55 shapes. We find that there is a link between the sequence-length-dependence of phenotype frequencies and differences in phenotypic robustness for deletions/insertions. In both parts of the plot, scatter points are plotted with some transparency, so that overlapping points can be distinguished, and the lines indicate a one-to-one correspondence (i.e. *x* = *y*).

Having analysed sequence-specific robustness variations for a fixed shape in the last section, we will now analyse shape-dependent robustness differences, i.e. differences in phenotype robustness. The data in figure 4*a* show that shapes with high phenotype robustness to substitutions also tend to have high phenotype robustness to insertions and deletions. Thus, neutral sets that are more robust to one type of mutation also tend to be more robust to another type of mutation.

Since phenotype robustness is measured as a fraction between zero and one for all types of mutations, we can compare the absolute robustness values for insertions and deletions. We hypothesize that the differences between these values could be linked to shape frequency changes: in principle, the fraction of sequences that fold into a given shape, the phenotype frequency as defined in [18], depends on the sequence length. It is possible that these changes in phenotypic frequency with sequence length are reflected in the difference between the robustness values to insertions and deletions. To test this, we computed a ‘shape robustness ratio’ for robustness to insertions compared to deletions. Figure 4*b* shows that this robustness ratio is correlated with the relative change in phenotype frequency from *L* + 1 to *L* − 1. Thus, comparing the absolute values of the phenotype robustness for insertions to that for deletions can indicate how a shape’s phenotype frequency changes with sequence length. Similar comparisons for substitutions versus deletions and insertions give similar results (data in electronic supplementary material).

### 3.3. Genotype non-neutral mutation probabilities

Non-neutral mutations, i.e. mutations causing a structural change, are quantified by the genotype mutation probability [18], ${\tilde{\phi }}_{q}^{\left(g\right)}$. ${\tilde{\phi }}_{q}^{\left(g\right)}$ quantifies the frequency of each non-neutral shape *q* in the mutational neighbourhood of a specific sequence *g* [18] (illustrated in figure 1). ${\tilde{\phi }}_{q}^{\left(g\right)}$ is therefore not a single value, but one value per structure *q* for each sequence *g*. Thus, a simple correlation analysis is not possible for ${\tilde{\phi }}_{q}^{\left(g\right)}$ variations. Instead, we adapt a method from Greenbury *et al.* [18]: they computed a similarity between ${\tilde{\phi }}_{q}^{\left(g\right)}$ and ${\tilde{\phi }}_{q}^{\left(h\right)}$ for fixed sequences *g* and *h*, taking into account all structures *q*, and compared this to a baseline similarity [18]. Here, we choose the Pearson correlation coefficient to quantify similarities because it is not affected by normalization factors.

**Figure 5.**
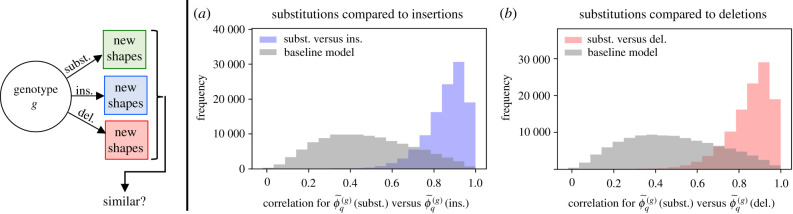
Genotype non-neutral mutation probabilities ${\tilde{\phi }}_{q}^{\left(g\right)}$: are there sequence-specific similarities between substitutions/deletions/insertions? (*a*) The similarity between ${\tilde{\phi }}_{q}^{\left(g\right)}$ (substitutions) and ${\tilde{\phi }}_{q}^{\left(g\right)}$ (insertions) is quantified by the Pearson correlation coefficient (blue data). In each comparison, the sequence *g* is kept fixed and the ${\tilde{\phi }}_{q}^{\left(g\right)}$ values are compared for all shapes *q*. As a baseline model, the analysis was repeated by comparing ${\tilde{\phi }}_{q}^{\left(A\right)}$ (subst.) of sequence *A* to ${\tilde{\phi }}_{q}^{\left(B\right)}$ (ins.) of another randomly chosen sequence from the same neutral set, *B* (grey values). Since the similarity is higher in the sequence-specific comparison than for the baseline model, the effects of different types of mutations are correlated, which we will summarize as generalized genetic correlations. (*b*) Similarly for ${\tilde{\phi }}_{q}^{\left(g\right)}$ (substitutions) and ${\tilde{\phi }}_{q}^{\left(g\right)}$ (deletions). Sequences from all neutral sets are used in the histograms, but only sequences with at least three non-neutral mutations in each of the mutational neighbourhoods by substitution, insertion and deletion were included.

${\tilde{\phi }}_{q}^{\left(g\right)}$ has so far been used only for substitutions. Here we include substitutions, insertions and deletions, i.e. three mutational neighbourhoods for each sequence. The approach described above allows us to compare the sequence-dependence of ${\tilde{\phi }}_{q}^{\left(g\right)}$ for different types of mutations. Specifically, we investigate whether the substitution neighbourhood of sequence *A* is more similar to the deletion neighbourhood of the same sequence than it is to the deletion neighbourhood of another sequence in the same neutral set. This analysis is performed for each sequence *g* in our sample and the data indicate that such similarities exist (figure 5*a*,*b*): mutational neighbourhoods by substitutions, insertions and deletions are more similar than expected from a baseline model that only accounts for average statistics of non-neutral variation. To ensure that results do not depend on using the Pearson correlation to quantify similarity, the analysis was repeated with alternative metrics with the same conclusions (data in electronic supplementary material). The observed similarities mean that a shape that is frequent in the substitution neighbourhood of a particular sequence is also likely to be frequent in the deletion and insertion neighbourhoods of the same sequence. This result extends the existing definition of genetic correlations [18], and we will therefore refer to this similarity as generalized genetic correlations.

Since the data in figure 5 summarized the data for sequences from all shapes, the next step is to ask if generalized genetic correlations exist if we separate these data by shape. For all shapes except one, we found that the correlation in the sequence-specific comparison was higher than in the baseline model for more than half the sequences in the sample (data in electronic supplementary material), indicating that generalized genetic correlations exist. The single exception is the stem-loop shape []. An explanation for this exception could be that highly robust sequences, like those in the neutral set of [], have a high number of neutral mutations and thus ${\tilde{\phi }}_{q}^{\left(g\right)}$ statistics are based on a small number of non-neutral mutations and have a higher variability. Alternatively, it is possible that the low number of stacks in shape [] is a structural reason for this exception.

To sum up, we found that shapes *q* that are frequent in the ${\tilde{\phi }}_{q}^{\left(g\right)}$ by substitution also tend to be frequent in the same sequence’s ${\tilde{\phi }}_{q}^{\left(g\right)}$ by deletion and insertion. Our baseline model shows that these similarities cannot simply be accounted for by arguing that certain shapes are frequent in all non-neutral variation from a specific neutral set. Therefore, we conclude that there are generalized genetic correlations.

### 3.4. Plastogenetic congruence for insertions and deletions

**Figure 6.**
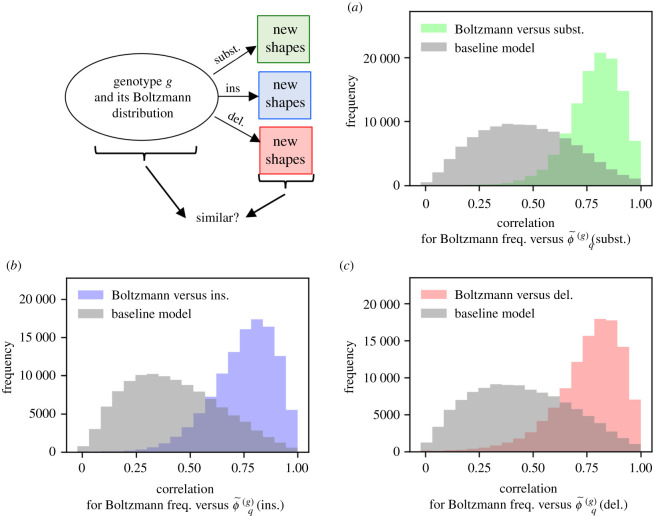
Genotype non-neutral mutation probabilities ${\tilde{\phi }}_{q}^{\left(g\right)}$: does plastogenetic congruence hold for all types of mutations? (*a*) The similarity between the genotype non-neutral mutation probability ${\tilde{\phi }}_{q}^{\left(g\right)}$ (substitutions) and the Boltzmann frequency $p_{q}^{\left(g\right)}$, both for shape *q* and sequence *g*, is quantified by the Pearson correlation coefficient (green data). As a baseline model, the analysis was repeated by comparing ${\tilde{\phi }}_{q}^{\left(A\right)}$ (subst.) of sequence *A* to $p_{q}^{\left(B\right)}$ of another randomly chosen sequence from the same neutral set, *B* (grey values). The similarity is lower in the baseline model and so there is a link between the effects of substitutions and the Boltzmann ensemble, i.e. plastogenetic congruence. (*b*,*c*) Analysis repeated for ${\tilde{\phi }}_{q}^{\left(g\right)}$ (insertions) and ${\tilde{\phi }}_{q}^{\left(g\right)}$ (deletions)—plastogenetic congruence exists in all cases. Sequences from all neutral sets are used in the histograms, but only sequences with at least three non-neutral mutations in each of the mutational neighbourhoods by substitution, insertion and deletion were included.

To understand our observations from a thermodynamic perspective, we build on Ancel and Fontana’s work on plastogenetic congruence [23]. They analysed non-neutral mutations in the special case of substitutions and found a similarity between the Boltzmann ensemble of a sequence and the frequent shapes obtained by mutations [23]. To understand non-neutral variation in our more general analysis, we test if the concept of plastogenetic congruence also applies to insertions and deletions. In our notation, plastogenetic congruence is the similarity between Boltzmann frequencies $p_{q}^{\left(g\right)}$ and ${\tilde{\phi }}_{q}^{\left(g\right)}$ for a specific sequence *g*. Therefore, plastogenetic congruence can be measured with the same approach as generalized genetic correlations in the previous section. The data in figure 6*a*–*d* show that the Boltzmann ensemble of a sequence is more similar to its mutational neighbourhood than in the baseline model for all three types of mutations. Again, the analysis was performed on a per-shape basis (data in electronic supplementary material) and plastogenetic congruence was found for all shapes except one. Therefore, we conclude that the principle of plastogenetic congruence holds for deletions and insertions as well as for substitutions for most shapes.

With this, we can build a consistent picture of mutual correlations between the Boltzmann ensemble and structural changes after mutations: the Boltzmann ensemble is a sequence-intrinsic property that does not depend on the type of mutation. This Boltzmann ensemble is linked to frequencies in all mutational neighbourhoods. In addition, there are correlations between these different mutational neighbourhoods, i.e. generalized genetic correlations.

### 3.5. Phenotype non-neutral mutation probabilities

In this final part, we will focus on non-neutral mutations averaged over neutral sets. These are quantified by the phenotype mutation probability *ϕ*_*qp*_ [3], as defined in figure 1: *ϕ*_*qp*_ is obtained by averaging the genotype mutation probability ${\tilde{\phi }}_{q}^{\left(g\right)}$ over all sequences *g* in the neutral set of shape *p* [18]. *ϕ*_*qp*_ is therefore a neutral-set average, just like phenotype robustness. Unlike phenotype robustness, however, *ϕ*_*qp*_ is not a single value: even for a fixed neutral set *p* there is one value per structure *q* to quantify the transition probability from *p* to *q* [3].

**Figure 7.**
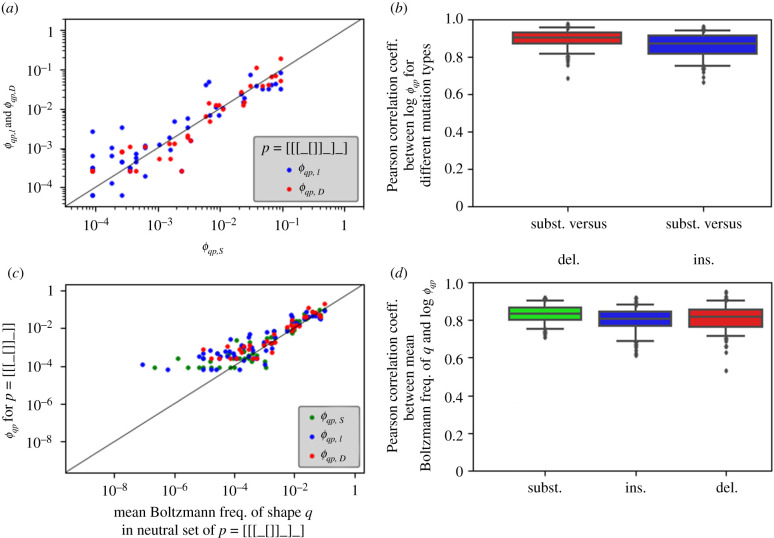
Phenotype mutation probabilities *ϕ_qp_* per neutral set *p*. (*a*) Phenotypic transitions to different new shapes *q*, starting from the neutral set of shape *p* = [[[_[]]_]_]: *ϕ*_*qp*,*I*_ (insertions, blue) and *ϕ*_*qp*,*D*_ (deletions, red) are plotted against *ϕ*_*qp*,*S*_ (substitutions). The line indicates a one-to-one correspondence. We find that the different types of *ϕ*_*qp*_ are correlated on a log–log scale and thus structural transitions that are likely to occur by substitutions also occur through insertions/deletions. (*b*) The calculations in (a) are repeated for all neutral sets p and summarized by the correlation coefficients between the log-log data. We find that correlations are high for all neutral sets. (*c*) Average Boltzmann frequency of shape *q* (computed over the neutral set of shape *p*) versus *ϕ*_*qp*_ for the neutral set of shape *p* = [[ [ []] ] ]. We find that the neutral-set-averaged Boltzmann frequencies are linked to *ϕ*_*qp*_. (*d*) The calculations in (c) are repeated for all neutral sets and summarized as correlation coefficients: for each shape *p*, Pearson correlation coefficients are calculated for the the logarithms of all non-zero values to quantify the correlation between *ϕ*_*qp*_ and mean Boltzmann frequencies of shape *q* in the neutral set of shape *p*. Again, correlation coefficients are high for all neutral sets. For the entire figure, Boltzmann frequencies, *ϕ*_*qp*,*S*_, *ϕ*_*qp*,*I*_ and *ϕ*_*qp*,*D*_ are computed from separate subsets of our sequence sample (125 sequences per neutral set for each calculation) to minimize the impact of generalized genetic correlations on our estimates of phenotypic quantities. Because of the limited number of sequences in the sample, low *ϕ*_*qp*_ values are likely to have some sampling error (as shown in the electronic supplementary material). Zero values are not shown due to the logarithmic scales.

Here, we calculate this mutation probability for deletions and insertions as well as for substitutions: these will be referred to as *ϕ*_*qp*,*D*_, *ϕ*_*qp*,*I*_ and *ϕ*_*qp*,*S*_. Figure 7*a* shows these mutation probabilities for all target shapes from a fixed initial neutral set of shape *p* = [[[_[]]_]_]. These values differ by several orders of magnitude and seem to be correlated linearly on the logarithmic scale. The analysis was repeated for the remaining neutral sets and the correlation coefficients are summarized in figure 7*b*. These correlation coefficients indicate that the correlations between *ϕ*_*qp*_ for the different types of mutations exist regardless of the chosen initial neutral set *p*.

Again the observed similarities can be placed in a larger context if we extend the arguments from the previous section: if plastogenetic congruence holds, shapes that tend to have high Boltzmann frequencies for sequences in the chosen neutral set are likely to have highest *ϕ*_*qp*_. Whether this neutral-set-wide plastogenetic congruence holds was tested by comparing neutral-set-averages of Boltzmann frequencies against the corresponding phenotypic mutation probabilities in figure 7*c*. We find that these two quantities are correlated for the example neutral set of shape *p* = [[[_[]]_]_]. Again, the analysis was repeated for all remaining neutral sets (figure 7*d*) and correlation coefficients were found to be high.

## 4. Discussion

### 4.1. Implications for evolutionary processes

First, we will discuss possible implications of the observed correlations for evolutionary processes. Existing research covers similar questions for substitutions, so we hypothesize how this would generalize. For substitutions, the genotype robustness of two mutational neighbours is often similar [7]. Such correlations can lead to differences in the rate of molecular evolution [44,50] and result in increasing robustness over the course of evolutionary processes [44]. We conjecture that the similarities in genotypic robustness for substitutions, insertions and deletions would enhance this overdispersion and increasing robustness. For non-neutral evolution, on the other hand, we would expect our generalized genetic correlations to amplify the effects of standard genetic correlations, which limit the diversity of accessible structural variation [18].

### 4.2. Implications for neutral set sizes

It has been shown for constant-length maps that inferences about neutral set topologies and neutral set sizes can be made from local mutational neighbourhoods [49,51]. In describing mutational neighbourhoods, we already touched on a similar link between phenotype robustness values and changes in phenotypic frequencies with sequence length. Future work could investigate neutral set sizes and how these change with sequence length in detail.

### 4.3. Establishing a more general sequence–structure map

More broadly, this analysis establishes a computationally feasible and biologically meaningful sequence–structure map that integrates multiple sequence lengths. Just like the RNA sequence–structure map became a model system, for which quantities like genotype and phenotype robustness and evolvability were established [6] before they were applied to a wider class of genotype–phenotype maps [52–54], this varying-length map could become a model system for establishing definitions that can be applied more broadly to varying-length genotype–phenotype maps.

In particular, one topic for further research is how neutral set sizes should be measured when the sequence length can vary. Neutral set size, or equivalently phenotype frequency, has been shown to be important for evolutionary outcomes in the constant-length case [3,55]. However, once the sequence length can change, the normalization factor, i.e. the number of all sequences of a given length, is sequence-length-dependent and therefore phenotype frequency and neutral set size are no longer interchangeable. Tian & Best [56] suggested neutral set size and phenotype frequency play two different roles, that of *designability* and that of *discoverability*. This hypothesis could be tested with simulations of evolutionary processes on this shapes map.

### 4.4. RNAshapes and the thermodynamic nature of sequence–structure maps

A further extension to the sequence–structure map framework could consider the full Boltzmann ensemble of each sequence. While individual Boltzmann probabilities are higher when the Boltzmann states are abstract shapes instead of detailed full secondary structures, a single shape cannot fully capture the folding space for a given sequence. Some previous work has already included more than one structure per sequence [9,12,23], but many of these studies consider evolutionary dynamics for one specific scenario, rather than quantifying sequence–structure map properties such as phenotypic frequencies, robustness and evolvability systematically for many structures. Establishing a general understanding of the sequence–structure map for this more general case will allow us to understand evolutionary processes for a variety of fitness functions, ranging from a riboswitch, where multiple shapes may be stabilized, to a single-fold molecule, where all suboptimal shapes are selected against.

### 4.5. Computational model

Like previous large-scale analyses of the RNA sequence–structure map, our analysis relies on computational structure predictions. Thus a caveat for our results is that secondary structure predictions are not perfect [48], like any computational model. For minimum-free-energy structures, changes to the model parameters have a small impact on general trends observed over large sequence samples [57]. If a similar argument holds in the shapes framework, our qualitative conclusions ought to generalize beyond the specific computational model.

## 5. Conclusion

In this analysis, we used the sequence–structure map of coarse-grained shapes because it can include sequences of different lengths within a single framework We extended existing definitions to quantify neutral and non-neutral mutations for insertions and deletions as well as for substitutions. With these definitions, we found multiple similarities between the structural effects of substitutions, insertions and deletions.

First, within each neutral set, sequences with high genotype robustness to substitutions also tend to have high genotype robustness to insertions and, for most shapes, to deletions. Second, phenotype robustness to substitutions is correlated with phenotype robustness to insertions and deletions. This means that some shapes are, on average, more robust to mutations than others and shapes with high phenotype robustness to substitutions also tend to have high phenotype robustness to insertions and deletions. Third, we found a generalized version of genetic correlations: any shape that is overrepresented in a sequence’s neighbourhood by substitution is likely to also be overrepresented in the neighbourhood by deletion and insertion. We showed that the principle of plastogenetic congruence applies to insertions and deletions as well as substitutions and forms a consistent picture with these generalized genetic correlations. Finally, we found correlations between insertions, deletions and substitutions for phenotype mutation probabilities *ϕ*_*pq*_. This means that if a transition from shape *A* to shape *B* is likely to happen by substitution, it is also likely to happen by insertion or deletion.

## Data accessibility

The code and data for this paper can be found at https://github.com/noramartin/insertions_deletions_RNA.
